# A Case Report of Puffer Fish Poisoning in Singapore

**DOI:** 10.1155/2013/206971

**Published:** 2013-12-04

**Authors:** Y. S. Yong, L. S. Quek, E. K. Lim, A. Ngo

**Affiliations:** Alexandra Hospital, Emergency Medicine Department, Singapore

## Abstract

Although many Asians regard puffer fish as a delicacy since ancient times, puffer fish (Lageocephalus scitalleratus) is also a well-known source of possibly lethal food poisoning. The fish is gaining popularity in Singapore and can be found in quite a few restaurants now. Puffer fish contains tetrodotoxin (TTX), a potent poison affecting the neural pathway. Puffer fish poisoning may cause a constellation of symptoms, such as giddiness, numbness and tingling sensation of the mouth, paresthesia, and muscle weakness. Severe cases may present with respiratory depression, circulatory failure, and death. TTX poisonings have been reported in Japan, Taiwan, Hong Kong, Bangladesh, and the United States (Haque et al. 2008). We report a case of mild poisoning and suggest observation for such cases.

## 1. Introduction

TTX is one of the most potent and oldest known neurotoxins, and puffer fish poisoning is common along the coasts of Asian countries [1–4]. TTX poisonings have been reported in Japan, Taiwan, Hong Kong, Cambodia [3], Bangladesh [4], and the United States. Also referred to as Fugu (meaning “river pig” in Japanese), balloonfish, blowfish, bubblefish, globefish, Patka fish, swellfish, toadfish, toadies, honey toads, sugar toads, and sea squab, the puffer fish [5] is commonly found in coastal regions of the tropics such as the Indian Ocean and in the South Pacific. They are relatively uncommon in the temperate zone and completely absent from cold waters [6].

Even with its long history of toxic effects, the fish is considered a delicacy in Japan especially and is prepared by licensed puffer fish cooks only in Japan. Despite this, reports of up to 50 deaths annually occur in Japan from puffer fish poisoning [7, 8]. In Taiwan, more than 100 cases were reported from 1998 to 2008, and the mortality rate was about 10% [9]. Ingestion of the flesh, viscera, or skin of toxic tetraodontiform fishes can cause poisoning. There are reports of a distinct relationship between gonadal activity of the fish and its toxicity, the fish being most lethal for consumption immediately prior to and during their reproductive periods [10, 11]. The highest concentration of the toxins is found in the viscera (gonads, especially the ovaries; liver; and intestine) and skin. The body musculature is usually free of poison [1]. To date, there is no known antidote available. Management remains supportive; hence people should be made aware of the potential risks of eating puffer fish, understand the symptoms and signs of poisoning, and quickly seek medical attention when such symptoms occur.

## 2. Case Report

A previously well 35-year-old Japanese lady with no significant past medical history presented to our emergency department with giddiness and weakness of the left upper limb and both lower limbs. She complained of numbness and tingling sensation around the mouth areas. There was no gastrointestinal symptoms. The symptoms occurred an hour after having fugu sashimi for lunch at a local Japanese restaurant. Her accompanying boyfriend has specified that they had ordered the gonads of the puffer fish, and these were cooked in hot soup. Apparently, they finished the meal because it was a high-end restaurant and food was described as very tasty. Her boyfriend who was with her at the restaurant had consumed less of the soup and was asymptomatic. Clinically, her parameters were stable. Heart rate was stable at 80 beats per minute and respiratory rate at 18 per minute. The patient remained conscious and alert throughout her consult and admission. On examination, she had decreased power over the left upper limb and both lower limbs. She was unable to get up from the bed to walk. A CT brain was done within the hour and this was normal. The electrocardiogram was normal, with no arrhythmias noted. Full blood count and electrolytes were also within normal limits. She was given oral stemetil initially for her nonspecific giddiness without relief of symptoms.

The patient was given activated charcoal and admitted for further observation. Her symptoms resolved the following day, after about 24 hours after ingestion of the fugu. She was discharged well and remained well following a telephone followup a week later. Although tetrodotoxin may be quantified in serum, whole blood, or urine to confirm poisoning, these tests are not available to us locally.

A report was subsequently filed with the National Environmental Agency, who then sent its team to do a check on the restaurant. Puffer fish is partially banned in Singapore. In Singapore, restaurants are allowed to import and serve only the flesh of the puffer fish, and special permits are required for this. Importation of the skin, gonads, and other parts of the fish is forbidden. The restaurant was found to be importing other parts of the puffer fish as well from Japan, including the gonads, and falsely declaring it and labeling it as other fish types.

## 3. Discussion

Puffer fish toxicity is a well-known type of fish poisoning. Puffer fish can be deadly if not prepared properly. Puffer fish poisoning toxicity results from consumption of incorrectly prepared puffer soup, fugu chiri, and sometimes from eating raw puffer meat, sashimi fugu. While chiri may be much more likely to cause death, sashimi fugu often causes intoxication, light-headedness, and numbness of the lips and is often eaten for this reason. The toxin cannot be deactivated by heat, cooking, or drying as it is heat stable and water soluble [12, 13].

Patients with puffer fish poisoning usually develop symptoms within 30 minutes to 6 hours of ingestion, with recovery usually in 24 hours [14, 15]. The duration, rapidity of onset, and severity of symptoms are dependant on the quantity of TTX consumed. Tetrodotoxin acts by blockage of the sodium channels of the heart myocytes, skeletal muscles, and the central and peripheral nervous systems. Tetrodotoxin also stimulates the chemoreceptor trigger zone in the medulla oblongata resulting in depression of the respiratory and vasomotor centres [16–18].

Clinical features include headache, diaphoresis, body numbness, dysarthria, dysphagia, nausea, vomiting, abdominal pain, generalized malaise, weakness, and lack of coordination. In more severe cases, hypotension, cardiac arrhythmias, muscle paralysis, and cranial nerve dysfunction may develop. Death results from respiratory failure and cardiovascular collapse and in severe cases can occur as early as 17 minutes after ingestion [19].

The progression of TTX poisoning is classified as follows.

 
*Grade 1.* Paresthesiae around the mouth, with mild gastrointestinal symptoms.
 
*Grade 2.* Paresthesiae spreading to the trunk and extremities, with early motor paralysis and lack of coordination.
 
*Grade 3.* Widespread paralysis, hypotension, and aphonia.
 
*Grade 4.* Impaired conscious state, respiratory paralysis, severe hypotension, and cardiac arrhythmia [20].

Diagnosis is usually made from medical history and toxidrome. Tetrodotoxin levels can help confirm diagnosis if history is ambiguous [21]. Diagnosis is mainly from patient's signs and symptoms in the presence of a positive history of puffer fish consumption or the detection of TTX in leftover food. If leftover food is unavailable, the determination of TTX in the patient's urine and/or plasma by mass spectrometry (MS) methods is essential to confirm the diagnosis [22].

Although various methods for the determination of TTX have been published, most of them are not available locally and in most countries. With better knowledge of the condition and good supportive care, the case-fatality rate was noted to have declined dramatically from 80% to 33% in 1974–1979 as compared to the early 20th century [23]. Patients surviving for more than 24 hours are believed to have good chances of recovery [24].

To date, there is no specific treatment for TTX poisoning and management remains largely symptomatic and supportive. Removal of unabsorbed toxin may be attempted by induced vomiting or gastric lavage or by giving activated charcoal to bind to any unabsorbed toxin. Since tetrodotoxin is less stable in an alkaline environment, instillation of 2% sodium bicarbonate has been suggested [1]. Cysteine has also been claimed to be effective in individual cases of puffer fish poisoning [25]. Other advocated treatment options have included cholinesterase inhibitors (neostigmine) [26], naloxone, and steroids. In animal studies, monoclonal antibodies and 4-aminopyridine have shown promising potential [27]. For mild cases like our patient, hospitals generally observe them for 24–48 hours and discharge when asymptomatic [28]. Patients with acute poisoning are symptomatic within 24 hours and usually recover without residual deficits although some take a few days to recover [29]. As such, it could also be recommended that mild cases may be observed in an observation unit in the emergency department for up to 24 hours and discharged if there is no progression of the symptoms. A telephone followup may be made the following day to ensure that the symptoms have resolved.

A few years back, there was a local newspaper report of a case of puffer fish poisoning by a local chef who was tasting imported fugu which his restaurant had bought and prepared in Japan with the toxins removed before export. In Japan, each prefectural local government has its own qualifying system for chefs, who are not allowed to serve fugu in other prefectures. Such a certification is not valid or available in Singapore [30]. Even with supposedly “nontoxic species” of puffer fish, there have been case reports of toxicity occurring [31]. The Japanese Ministry of Health and Welfare (presently the Ministry of Health, Labour, and Welfare) published a guideline for edible puffer fish in 1983, with updates in 1993 and 1995 [32–34]. In Taiwan and China, consumption of puffer fish is banned [35]. In Singapore, puffer fish is becoming popular, and currently there is no available method for testing for TTX in biological samples.

In Singapore, healthcare providers are not required to report such illnesses. Due to underrecognition, milder cases may remain undiagnosed or unreported; hence the actual number of poisonings may be much greater. There are about 30 cases of poisoning by marine toxins reported in the United States annually. It is estimated from cases with available data that one person dies every 4 years from toxic seafood poisonings [36].

## 4. Conclusion

The objective of the case report is to highlight recognition and management of puffer fish poisoning. A dietary history is important, which is often neglected in many busy accident and emergency departments.

Mild cases may be observed in the emergency department and discharged with a telephone followup if the symptoms do not progress after 24 hours. We would like to recommend that rapid notification of public health authorities be made essential. This will allow timely investigation by the relevant authorities to identify the source of contaminated seafood and prevent further poisonings [29]. A food and dietary history is important, especially in younger patients who present with nonspecific neurological signs and symptoms after a meal. Health professionals should be aware of the condition so as to institute early and appropriate management.
